# Calcium Buffering in Astrocytes and Its Relevance for Experimental Data Interpretation and Computational Modeling

**DOI:** 10.1111/jnc.70470

**Published:** 2026-06-04

**Authors:** Kerstin Lenk, Andre Zeug, Franziska E. Müller

**Affiliations:** ^1^ Institute of Neural Engineering Graz University of Technology Graz Austria; ^2^ BioTechMed Graz Austria; ^3^ Hannover Medical School Institute of Neurophysiology, Cellular Neurophysiology Hannover Germany

**Keywords:** astrocytes, buffering, calcium, computational modeling

## Abstract

Astrocytic Ca^2+^ signaling is essential for maintaining physiological brain function, including the modulation of synaptic transmission, neurovascular coupling, and ion homeostasis. However, the spatiotemporal dynamics of astrocytic Ca^2+^ activity are highly sensitive to Ca^2+^ buffering, which shapes the amplitude, duration, and spread of cytosolic and organellar signals. These buffers include endogenous components such as cytosolic Ca^2+^ binding proteins, as well as organelles like the endoplasmic reticulum acting as Ca^2+^ stores. Additionally, exogenous buffers are introduced in experiments, including chelators, synthetic dyes, and genetically encoded Ca^2+^ indicators. Both types of buffers can profoundly alter experimental observations, making it challenging to accurately interpret Ca^2+^ dynamics. Computational modeling offers a powerful approach to separate these effects, enabling systematic exploration of how the buffering capacity of specific system components influences astrocytic intracellular and intercellular signaling. By incorporating experimental data with realistic biophysical buffering parameters, models can make predictions that are difficult to achieve empirically and help identify key parameters that shape astrocytic Ca^2+^ physiology. In this review, we discuss how buffering components influence astrocyte Ca^2+^ activity and their integration into modeling predictions. Future advances in computational modeling, combined with extensive experimental data, will be crucial for enhancing our understanding of astrocytic Ca^2+^ regulation and elucidating its role in health and disease.

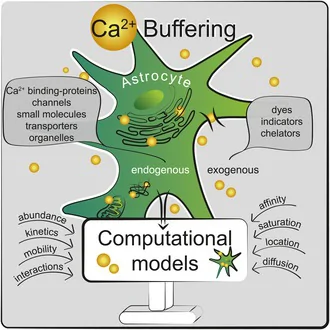

## Graphical abstract

Components and properties of Ca^2+^ buffers in astrocytes, which should be considered for data interpretation and in computational modeling of astrocyte Ca^2+^ activity.

**Abbreviations**
AC: adenylyl cyclase
AMPAR: α‐amino‐3‐hydroxy‐5‐methyl‐4‐isoxazolepropionic acid receptor
ATP: adenosine triphosphate
B: buffer molecule
Ca^2+^
: calcium
CaM: calmodulin
CaMKII: Ca^2+^–calmodulin‐dependent protein kinase II
cAMP: cyclic adenosine monophosphate
ChR2: Channelrhodopsin‐2
EC_50_
: half‐maximal effective concentration
ECM: extracellular matrix
EET: epoxyeicosatrienoic acid
EM: electron microscopy
ER: endoplasmic reticulum
GECI: genetically encoded Ca^2+^ indicators
H^+^
: hydrogen
iGluR: ionotropic glutamate receptor
IP_3_
: inositol trisphosphate
IP_3_R: IP_3_ receptor
K^+^
: potassium
KAR: kainic acid receptor
*K*_d_
: dissociation constant
*K*_d,app_
: effective association rate
*k*_off_
: dissociation rate
*k*_on_
: association rate
MCU: mitochondrial Ca^2+^ uniporter
Mg^2+^
: magnesium
Na^+^
: sodium
NCLX: mitochondrial Na^+^/Ca^2+^ exchanger
NCX: Na^+^/Ca^2+^ exchanger
NMDAR: N‐methyl‐D‐aspartate receptor
NVC: neurovascular coupling
NVU: neurovascular unit
ODE: ordinary differential equation
Orai1: Ca^2+^ release‐activated Ca^2+^ modulator 1
P2X: Purinergic receptor
PM: plasma membrane
PMCA: plasma membrane Ca^2+^‐ATPase
RyR: ryanodine receptors
SERCA: sarco/ER Ca^2+^‐ATPase
SOCE: store‐operated Ca^2+^ entry
STIM1: Stromal interaction molecule 1
TRP: transient receptor potential
TRPV4: transient receptor potential vanilloid 4
VGCC: voltage‐gated Ca^2+^ channel

## 1 Introduction

Buffering is the process that helps maintain conditions within a range that supports proper function despite fluctuations. Ion buffering across the entire brain is crucial for preserving the delicate balance of extracellular ions, which is essential for proper network function. Within this larger system, astrocytes play a key role by regulating local ion concentrations. The entire network of interconnected astrocytes, known as the astrocyte syncytium, functions as a critical buffering system. This glial cell type modulates potassium (K^+^) concentrations through the expression of various voltage‐dependent and ‐independent channels and is further essential in regulating sodium (Na^+^) levels. Most notably, astrocytes exhibit highly complex and polymorph fluctuations in calcium (Ca^2+^) concentrations within their cytosol. These Ca^2+^ dynamics are regarded as a special form of signaling, contributing overall to brain homeostasis and intercellular communication, as Ca^2+^ is a major second messenger (Khakh and McCarthy 2015; Rusakov 2015; Semyanov et al. 2020; Veiga et al. 2025). Furthermore, astrocytes not only modulate neuronal signaling but also coordinate vascular responses and influence synaptic plasticity, which heavily rely on tightly controlled intracellular Ca^2+^ dynamics (Bazargani and Attwell 2016; Carmignoto and Gómez‐Gonzalo 2010; Henneberger et al. 2010; Lia et al. 2023; Perea and Araque 2007; Sanz‐Gálvez et al. 2024; Sasaki et al. 2014).

Typical astrocytic resting Ca^2+^ concentrations range from 50 to 120 nM in the cytosol (King et al. 2020; Shigetomi et al. 2012). In contrast, concentrations within internal organelles—such as the endoplasmic reticulum (ER) and mitochondria—can reach several hundred micromolar, creating significant gradients for rapid signaling and sequestration. All reported concentrations depend on various factors, including experimental techniques, the brain region studied, preparation methods, species, and temperature. The resting Ca^2+^ concentration in astrocytes has been reported to change during development and under pathological conditions (Kuchibhotla et al. 2009; Zheng et al. 2015).

Ca^2+^ buffering involves mechanisms by which cells stabilize the concentration of free Ca^2+^ ions in their cytosol, ensuring that intracellular signaling and homeostasis are maintained accurately (for a detailed definition of Ca^2+^ sensing and buffering, see Schwaller (2010)). Less than 1% of a cell's Ca^2+^ ions exist as free Ca^2+^ in the cytosol (Eisner et al. 2023). Still, this small amount is crucial for regulating essential physiological processes, from the release of gliotransmitters shaping synaptic transmission to the regulation of enzymes and gene expression. Ca^2+^ buffers represent a potent mechanism by which the spread of astrocytic intracellular Ca^2+^ signals could be controlled (Wang et al. 1997).

Immobile Ca^2+^ buffers are structural proteins or molecules with nearly zero diffusion coefficients (Matthews and Dietrich 2015). Examples include cytoskeleton‐associated proteins, such as glial‐specific S100β, and unidentified fixed binding sites. Mobile buffers are diffusible molecules, including endogenous proteins and exogenous chelators. Naturally occurring molecules, like calcium‐binding proteins such as calreticulin, parvalbumin, and calmodulin, determine the magnitude and dynamics of buffering through their Ca^2+^ affinities and kinetics (reviewed in e.g., Eisner et al. 2023; Haiech et al. 2019; Schwaller 2010). Additionally, organelles such as the ER and mitochondria act as dynamic Ca^2+^ stores and buffers, directly influencing cytosolic Ca^2+^ availability. Physiologically, these buffers modulate both the amplitude and temporal profile of Ca^2+^ signals through uptake and release mechanisms.

Exogenous buffering can arise from the application of synthetic Ca^2+^ chelators during experiments, such as BAPTA and EGTA, or from genetically encoded Ca^2+^ indicators (GECI; e.g., GCaMPs) and dyes (e.g., Fura‐2, Fluo‐4) used to visualize astrocyte Ca^2+^ activity. These tools, while invaluable for quantifying intracellular Ca^2+^, can themselves alter cellular Ca^2+^ dynamics by introducing additional buffering capacity, hence complicating data interpretation in both experimental and computational settings (Semyanov et al. 2020; Wang et al. 1997).

Fundamentally, the capacity of Ca^2+^ buffers depends on several interrelated mechanisms, including (i) affinity, (ii) kinetics, (iii) concentration, (iv) mobility and distribution, (v) saturation states, and (vi) cooperative or competitive binding possibilities. Buffer proteins differ in how tightly and how rapidly they bind Ca^2+^, influencing both their sensitivity and responsiveness to Ca^2+^. Additionally, the abundance and location of buffer proteins determine the spatial aspect of buffering. Moreover, highly mobile versus immobile buffers can shape either local Ca^2+^ microdomains or global dynamic hotspots. During large Ca^2+^ fluxes, buffering capacity can be saturated, and in some cases, buffers bind Ca^2+^ cooperatively, further modulating signal dynamics (Naraghi and Neher 1997).

Defective intracellular Ca^2+^ regulation is associated with numerous disease conditions, with abnormal Ca^2+^ buffering being a common underlying cause (Bancroft and Srinivasan 2022; Shah et al. 2022; Shigetomi et al. 2016; Verkhratsky et al. 2019). This dysregulation contributes to neurodegenerative diseases such as Parkinson's and Alzheimer's, where abnormal astrocytic Ca^2+^ activity leads to neuronal dysfunction and disease progression (Bancroft and Srinivasan 2022). Chronic Ca^2+^ overload from faulty buffering can trigger harmful pathways, like excessive calcineurin signaling and neuroinflammation, which worsen injury and cellular stress (Lim et al. 2023). In astrocytes, disruptions in endogenous Ca^2+^ buffering or changes caused by experimental probes can significantly skew data interpretation—masking or overstating accurate physiological Ca^2+^ kinetics. Computational models must therefore account for all sources of buffering to simulate in vivo Ca^2+^ dynamics and avoid incorrect predictions accurately. Ultimately, understanding Ca^2+^ buffering capacity in astrocytes is essential for designing reliable experiments and developing precise models of glial function in health and disease.

In recent years, interest in computational models has increased as they predict the spread, intensity, and duration of events like neurotransmitter release and changes in ion dynamics (De Pittà 2022; Lenk et al. 2024; Manninen et al. 2018; Oschmann et al. 2018; Rusakov 2015). These models aim to understand and predict astrocyte function as well as the brain's information‐processing capability. They partly rely on published experimental results, incorporating ion concentrations and diffusion constants, and unique cellular properties such as cell morphology and organelle distribution. Importantly, these models often overlook that about 99% of Ca^2+^ ions are not freely dissolved in the cellular milieu but are reversibly bound to Ca^2+^ buffers. Therefore, it is essential to include aspects of Ca^2+^ buffering in computational models used to analyze cellular function. In this context, they may support the accurate prediction of disease onset and progression, as well as identify potential rescue strategies tied to aberrant astrocyte Ca^2+^ signaling.

**FIGURE 1:**
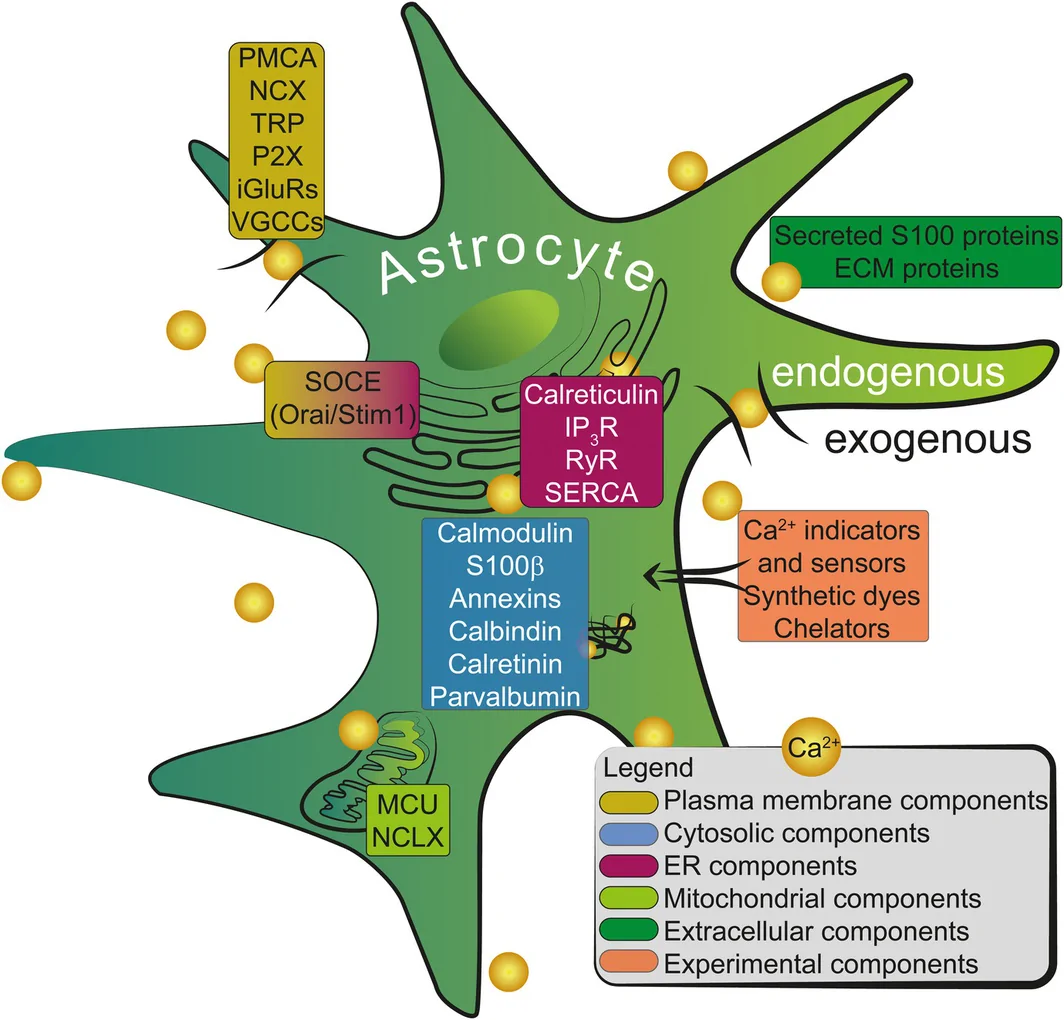
Basic summary of the components relevant for Ca^2+^ buffering in astrocytes. Endogenous buffers include cytosolic, mitochondrial, and ER contributors, and channels and transporters govern Ca^2+^ transport through the plasma membrane, where extracellular proteins regulate Ca^2+^ availability. Experimentally introduced buffers further shape observed Ca^2+^ dynamics. ECM, Extracellular matrix; ER, Endoplasmic reticulum; iGluRs, Ionotropic glutamate receptors; IP_3_R, Inositol trisphosphate receptor; MCU, Mitochondrial Ca^2+^ uniporter; NCLX, Mitochondrial Na^+^/Ca^2+^ exchanger; NCX, Na^+^/Ca^2+^ exchanger; P2X, Purinergic receptor; PMCA, Plasma membrane Ca^2+^‐ATPase; RyR, Ryanodine receptors; SERCA, Sarco/ER Ca^2+^‐ATPase; SOCE, Store‐operated Ca^2+^ entry; TRP, Transient receptor potential; VGCCs, Voltage‐gated Ca^2+^ channels.

This review, therefore, focuses on the role of Ca^2+^ buffers influencing astrocytes (Figure 1), including both physiological endogenous buffers and synthetic exogenous buffers, which impact experimental results used in computational simulations. We summarize how current models incorporate Ca^2+^ buffering effects so far and offer perspectives on potential future directions, aiming to provide valuable suggestions for improving computational modeling of astrocyte Ca^2+^ dynamics.

## 2 Physiological Ca^2+^ Buffering in Organic Matter

Endogenous buffering describes the regulation through molecules and proteins that bind Ca^2+^ ions, thereby regulating the concentration and movement of free Ca^2+^ within the cytosol. Since Ca^2+^ is a common second messenger, buffering is essential for shaping Ca^2+^‐mediated signaling (Berridge et al. 2003). This is especially relevant in astrocytes, which exhibit unique patterns of Ca^2+^ events within their cytosol (Khakh and McCarthy 2015; Semyanov et al. 2020; Verkhratsky and Nedergaard 2018; Volterra et al. 2014). These intracellular events can be described as polymorphic because they vary in size, duration, travel distance, magnitude, point of origin, and more. Accordingly, many names have been proposed to reflect the different types of Ca^2+^ events, including puffs, blips, oscillations, waves, sparkles, bursts, flares, and more (Khakh and McCarthy 2015; Pasti et al. 1997; Swillens et al. 1999).

The characteristics of astrocyte Ca^2+^ events are shaped by their intracellular site of occurrence: Astrocytes typically show low‐frequency (∼0.01–0.03 Hz), long‐lasting (∼5–15 s), high‐magnitude (≥ 1.5 fold‐change) Ca^2+^ events in the cytosol of their big somatic volume, while in microdomains distant from the soma, that is, in fine processes, high‐frequency (∼0.7 Hz), rapid (∼0.5–5 s) and small magnitude (≤ 1.5 fold‐change) events dominate (Agarwal et al. 2017; Chen, Ye, et al. 2025). Importantly, these values will differ between astrocytes from different brain regions and depend on the preparation and experimental conditions.

For these multiform Ca^2+^ events to occur, astrocytes require a distinct molecular machinery that supports slower uptake, release, and intracellular buffering of Ca^2+^, different from that in neurons, where free Ca^2+^ must be rapidly removed from the cytosol to terminate fast electrical signaling and prevent excitotoxicity. The Ca^2+^ signaling of astrocytes requires a distinct set of Ca^2+^ channels, transporters, Ca^2+^‐binding proteins, and organelles that enable astrocytes to generate prolonged, localized Ca^2+^ elevations.

### 2.1 Kinetics, Affinity, and Capacity

Cytosolic Ca^2+^ events occur either from the influx of extracellular Ca^2+^ or from the release of Ca^2+^ stored within intracellular compartments, or a combination of both, leading to fluctuations in free cytosolic Ca^2+^ (Semyanov et al. 2020). To understand how Ca^2+^ buffers affect cellular Ca^2+^ signaling, it is important to consider both the speed (kinetics) and strength of Ca^2+^ binding (affinity) (Keener and Sneyd 2025). The binding of Ca^2+^ to a buffer molecule (*B*) is controlled by two rates: the association rate (*k*_on_), which shows how quickly Ca^2+^ ions bind to the buffer, and the dissociation rate (*k*_off_), which indicates how fast they are released:

(1) $$\mathrm{Ca}+B\begin{array}{c} k_{\mathrm{on}}\\ ⇌\\ k_{\mathrm{off}} \end{array}\mathrm{CaB}$$

The equilibrium between these two rates defines the dissociation constant (*K*_d_), which is calculated as *K*_d_ *= k*_off_ */k*_on_· *K*_d_ indicates the concentration of free Ca^2+^ at which half of the buffering sites are occupied, and thus reflects the buffer's affinity for Ca^2+^. A lower *K*_d_ denotes higher affinity.

Typically, Ca^2+^ binds to buffers extremely rapidly, often at rates close to the limit set by molecular diffusion (Eigen 1963). Determining these rates experimentally is difficult; therefore, *k*_off_ is usually measured directly, while *k*_on_ is inferred from the known *K*_d_.

The effective affinity of a buffer is not static but depends on the cellular milieu. Many Ca^2+^‐binding proteins lack strict selectivity, so additional intracellular ions, such as magnesium (Mg^2+^) and hydrogen (H^+^), directly compete with Ca^2+^ at shared sites, where they occupy these sites and thereby lower the apparent affinity and effective buffering capacity for Ca^2+^.

When Mg^2+^ or H^+^ are already bound, they must dissociate before Ca^2+^ can attach, adding an extra kinetic step that slows the overall *k*_on_ of Ca^2+^ with the buffer. This reduction in the effective association rate (*K*_d,app_) appears as a reduced apparent affinity for Ca^2+^, even though the buffer's intrinsic chemical affinity for Ca^2+^ remains the same. Assuming a single binding site which is accessible for various ions, the equilibrium concentration of Ca^2+^ bound to the buffer [*CaB*], will be given by

(2) $$\left[\mathrm{CaB}\right]={\left[B\right]}_{\mathrm{Tot}}\cdot \frac{{\left[{\mathrm{Ca}}^{2+}\right]}_{i}}{K_{d,\mathrm{app}}+{\left[{\mathrm{Ca}}^{2+}\right]}_{i}},$$

with $K_{d,\mathrm{app}}=K_{d,\mathrm{Ca}}\cdot \left(1+\frac{{\left[{\mathrm{Mg}}^{2+}\right]}_{i}}{K_{d,\mathrm{Mg}}}+\frac{{\left[H^{+}\right]}_{i}}{K_{d,H}}\right)$ and $K_{d,\mathrm{Ca}}$, $K_{d,\mathrm{Mg}}$, and $K_{d,H}$ as the equilibrium dissociation constants, for Ca^2+^, Mg^2+^, and H^+^, respectively, such that *K*_d,Ca_ *= k*_off,Ca_ */k*_on,Ca_, and similar for *K*_d,Mg_ for *K*_d,H_. A comprehensive elaboration can be found in the work of Eisner et al. (2023).

The magnitude of this effect varies widely among the different astrocytic Ca^2+^‐binding proteins. In astrocytes, essential buffering proteins, such as those from the S100 family (e.g., S100β) and annexins, have EF‐hand and annexin‐type Ca^2+^‐binding domains, respectively, which can also bind Mg^2+^ and be influenced by proton concentration. Mg^2+^ binding to these proteins stabilizes them in conformations distinct from their Ca^2+^‐bound states, potentially modulating their sensitivity and responsiveness to Ca^2+^ signaling. Protons influence Ca^2+^ binding through pH‐dependent changes in buffer ionization and binding site availability, with acidic conditions generally lowering Ca^2+^ affinity. Therefore, the dynamic interaction among Ca^2+^, Mg^2+^, and protons in astrocytes can determine the spatial and temporal features of intracellular Ca^2+^ signals. This interplay alters how astrocytic buffers shape both the amplitude and kinetics of Ca^2+^ signaling in physiological and pathophysiological contexts (Grabarek 2011).

In astrocytes, cooperative binding of Ca^2+^ to buffer proteins plays an important role in shaping the dynamics of intracellular Ca^2+^ signaling. Positive cooperativity occurs when the binding of one Ca^2+^ ion to a buffer protein increases the affinity of its remaining binding sites for additional Ca^2+^ ions. This effect is seen in some astrocytic Ca^2+^‐binding proteins that contain multiple EF‐hand motifs or other multi‐site binding domains (Nelson et al. 2002). For these proteins, an initial Ca^2+^ binding event causes structural changes that make subsequent binding easier, resulting in a more sensitive and nonlinear response to rising intracellular Ca^2+^ levels. This positive cooperativity enhances Ca^2+^ buffering and increases overall affinity (lowers *K*_d_). Additionally, positive cooperativity helps astrocytes propagate strong and localized Ca^2+^ transients, especially within fine processes where spatial constraints require efficient buffering and signaling.

Negative cooperativity, on the other hand, happens when Ca^2+^ binding to one site decreases the affinity at other sites on the same protein. In principle, such a mechanism could modulate the response of a buffer to rising Ca^2+^ levels to limit excessive or prolonged Ca^2+^ binding and thereby stabilize local Ca^2+^ signals. In astrocytes, however, negative cooperativity has not been firmly established as a common property of endogenous Ca^2+^‐binding proteins and is currently best viewed as a possible, but not yet well‐documented, contributor to the shaping of Ca^2+^ transients (Gifford et al. 2007).

Together, these cooperative binding mechanisms provide astrocytic buffers with the capacity not only to bind Ca^2+^ but also to finely regulate the sensitivity and kinetics of Ca^2+^ transients. This adjustment supports various physiological functions, from modulating neurotransmitter release to the regulation of blood flow, emphasizing the adaptability of astrocytic Ca^2+^ signaling machinery to the complex demands of the brain environment (Denizot et al. 2019; Iida and Potter 1986; Lia et al. 2021).

A precise distinction in Ca^2+^ buffering formalism is between the buffer capacity *κ* and the buffering power *β*. The buffer capacity *κ* is defined as the differential ratio (local slope) of bound to free Ca^2+^, $\kappa =d\left[{\mathrm{Ca}}_{B}\right]/d\left[{\mathrm{Ca}}^{2+}\right]$. For a single fast, reversible buffer at quasi‐equilibrium it equals $\left[B_{\mathrm{tot}}\right]\cdot K_{d}/{\left(K_{d}+\left[{\mathrm{Ca}}^{2+}\right]\right)}^{2}$, and the total endogenous capacity is obtained by summing the individual contributions of each buffer species, so that $\kappa_{\text{total}}=\sum_{i}\kappa_{i}$ (see Eisner et al. 2023). In astrocytes, multiple endogenous proteins contribute to this sum, and exogenous indicators add additional terms when present; because binding saturates, *κ* varies with the instantaneous free Ca^2+^ concentration (Naraghi and Neher 1997; Zhou and Neher 1993). The buffering power *β* relates changes in total cytosolic Ca^2+^ to changes in free Ca^2+^, $\beta =d{\left[\mathrm{Ca}\right]}_{\text{total}}/d\left[{\mathrm{Ca}}^{2+}\right]$, and under the rapid buffer approximation—when fast‐binding buffers remain near equilibrium with free Ca^2+^—it equals $1+\kappa_{\text{total}}$. Biophysically, higher *κ* (and thus higher *β*) means that endogenous buffers sequester a larger fraction of the Ca^2+^ load, so peaks in free Ca^2+^ are smaller and the apparent kinetics of free Ca^2+^ become slower. Under the rapid‐buffer approximation, these immobile endogenous buffers primarily act locally: they reduce the effective diffusion of free Ca^2+^ and help confine Ca^2+^ microdomains, thereby limiting spatial spread while still allowing fast local shaping of signals. In contrast, mobile buffers redistribute through the cytoplasm and produce related slowdowns in amplitude and kinetics, but in a manner that depends on both their binding rates and their diffusion, allowing them to transport Ca^2+^ away from the source and modulate more extended spatial profiles (Naraghi and Neher 1997; Wagner and Keizer 1994). Strictly speaking, sequestration by the ER and mitochondria is better represented as separate uptake and release fluxes on slower time scales rather than as part of *κ*; however, over short intervals, these organelles can behave like additional, slow buffering components that further prolong recovery of free Ca^2+^ (Neher and Augustine 1992).

**FIGURE 2:**
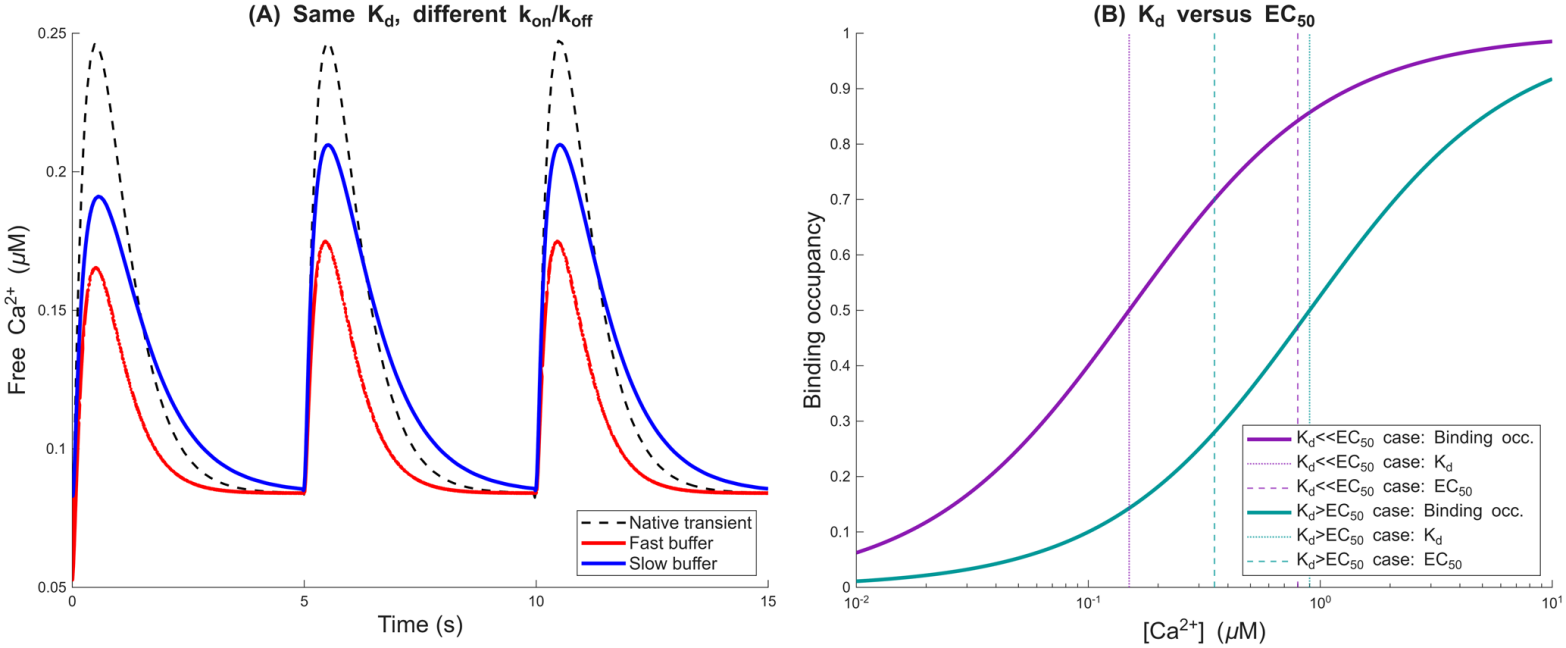
Astrocyte Ca^2+^ buffering and indicator readout schematics. (A) Effect of buffer kinetics on an oscillatory astrocytic Ca^2+^ transient when buffer affinity is kept constant. The “native” free Ca^2+^ signal (black dashed) is modeled as a repetitive alphalike transient around a resting [Ca^2^^+^] of 0.084 μM, consistent with astrocytic baseline values reported by Shigetomi et al. 2016. The cell contains either no cytosolic buffer, a fast buffer, or a slow buffer, all with the same dissociation constant *K*_d_ = 50 μM (low‐affinity regime) and total concentration *B*_*tot*_ = 30 μM; only *k*_on_ and *k*_off_ differ, so that fast binding/unbinding sharply attenuates and confines the Ca^2+^ peaks, whereas slow binding/unbinding permits larger peak [Ca^2+^] and slower recovery. (B) Conceptual separation between binding affinity (*K*_d_) and optical readout sensitivity (EC_50_) for Ca^2+^ indicators. Y‐axis: Binding occupancy (θ) of two hypothetical indicators across [Ca^2+^] (0.01–10 μM) for a “high‐affinity/low sensitivity” sensor (purple; *K*_d_ = 0.15 μM; “K_d_ ≪ EC_50_” case) and a “lower‐affinity/high sensitivity” sensor (cyan; *K*_d_ = 0.90 μM; “K_d_ > EC_50_” case). Vertical dotted lines mark *K*_d_ and solid lines mark EC_50_ for each indicator, highlighting that the Ca^2+^ concentration for half‐maximal binding (*K*_d_) and half‐maximal fluorescence (EC_50_) can diverge in either direction. The MATLAB code to produce the figure can be found here: https://github.com/kerstinlenk/calciumbuffering.

The interaction of multiple astrocytic buffers with different affinities and kinetics creates a dynamic and precisely tuned buffering environment. This environment regulates the development of spatial Ca^2+^ gradients, the propagation of signals through the astrocytic arbor, and the coordination and influence of astrocytes on neuronal activity and blood flow regulation. The kinetics of Ca^2+^ transients in astrocytes are heavily affected by the binding rates *k*_on_ and *k*_off_ of Ca^2+^ buffers, even if their affinity (*K*_d_) is similar. Although two buffers may have comparable overall Ca^2+^ binding strength, differences in how quickly they bind and release Ca^2+^ can significantly change the shape and timing of Ca^2+^ signals (Wang et al. 1997). This is illustrated in Figure 2A, where three buffers with identical *K*_d_ but different *k*_on_ and *k*_off_ reshape a stereotyped astrocytic Ca^2+^ transient in distinct ways.

A buffer with high *k*_on_ quickly binds free Ca^2+^ during a transient, effectively reducing the peak amplitude of the Ca^2+^ spike. When paired with moderate or fast *k*_off_, it can release Ca^2+^ rapidly, allowing the transient to decay more quickly. This leads to sharper, shorter Ca^2+^ signals. Such kinetics are essential in astrocyte fine processes (like perisynaptic astrocytic processes), where fast, localized Ca^2+^ buffering helps regulate rapid, spatially confined gliotransmission events (Lia et al. 2021). S100β proteins, for example, which are abundant in astrocyte processes, exhibit rapid binding kinetics suited to these fast Ca^2+^ microdomains (Van Eldik et al. 1984).

Conversely, a buffer with a slower *k*_on_ binds Ca^2+^ more gradually, allowing a higher peak of free Ca^2+^ during the transient. With a slow *k*_off_, Ca^2+^ remains bound longer, prolonging the decay phase of the transient and smoothing out fluctuations over time. Buffers of this type contribute to slower, sustained Ca^2+^ signals typically seen in the astrocyte soma or larger branches, where signals integrate over broader spatial and temporal domains. For instance, annexin family proteins tend to have slower Ca^2+^ binding and release rates, modulating slower, global Ca^2+^ waves (Hermann et al. 2012; Patton et al. 2025).

The buffering power (reflecting both the concentration of these buffers and their affinities) further controls transient kinetics by determining how much free Ca^2+^ is sequestered overall. High buffering power dampens the amplitude and prolongs the duration of Ca^2+^ transients by trapping more Ca^2+^ and slowing its clearance (Neher and Augustine 1992; Sasaki et al. 2014; Wang et al. 1997).

Thus, the combination of kinetics and buffering power of diverse astrocyte buffers, distributed differentially across subcellular compartments, allows fine control of the amplitude, duration, and spatial spread of intracellular Ca^2+^ signals. This kinetic diversity underlies the adaptability of astrocytes in responding to varied physiological stimuli and controlling neuron–glia interactions (Khakh and McCarthy 2015). Table 1 summarizes the ranges of the resting Ca^2+^ concentrations in the cytosol, ER, mitochondria, and the extracellular space.

**TABLE 1:** Ranges of resting Ca^2+^ concentrations in astrocytes.

Compartment	Total [Ca^2+^]	Free [Ca^2+^]	Role in astrocyte Ca^2+^ signaling	References
Cytosol	~100–200 μM (total, bound & free), increasing gradient towards periphery	~50–120 nM (resting); peaks up to 500 nM–1 μM during transients	Multifarious Ca^2+^ transients: slow propagating events and rapid Ca^2+^ transients and oscillations; local microdomains in processes; coupling to gliotransmitter release and metabolic pathways	Aryal et al. (2022), King et al. (2020), O'Connor and Kimelberg (1993), Parpura and Haydon (2000), Romagnolo et al. (2024), Shigetomi et al. (2012, 2016)
ER	1–5 mM (total, bound mainly by calreticulin & other luminal proteins)	~100–500 μM free Ca^2+^	Major intracellular Ca^2+^ store; IP_3_R and RyR‐mediated release drive Ca^2+^ oscillations and intercellular Ca^2+^ waves; SERCA refills stores	Aryal et al. (2022), Michalak (2024), Parpura et al. (2011)
Mitochondria (matrix)	~0.5–1 mM (total, bound)	Resting ~100–200 nM; rises to μM levels near microdomains during ER/PM release	Act as dynamic Ca^2+^ buffers; shape local microdomain Ca^2+^; regulate ATP production; protect against cytosolic overload	Boitier et al. (1999), Rizzuto et al. (2012)
Extracellular space (brain interstitial fluid)	~1.3–1.8 mM free Ca^2+^	Essentially all free (few buffers, but ECM & proteins bind some)	Driving force for Ca^2+^ influx through channels (TRP, P2X, SOCE); modulated by neuronal/glial activity; influences excitability and astrocytic signaling	Nicholson et al. (1978), Rusakov and Fine (2003)

Abbreviations: ATP, adenosine triphosphate; ECM, extracellular matrix; ER, endoplasmic reticulum; IP_3_R, inositol trisphosphate receptor; PM, plasma membrane; RyR, ryanodine receptors; SERCA, sarco/ER Ca^2+^‐ATPase; SOCE, store‐operated Ca^2+^ entry; TRP, transient receptor potential.

### 2.2 Proteins, Organelles, Transporters

Within the lumen of astrocytic intracellular stores, a substantial amount of Ca^2+^ is buffered by Ca^2+^‐binding proteins, such as calreticulin. These organellar proteins differ from cytosolic Ca^2+^‐binding proteins, such as parvalbumin, calbindin, and the S100 protein family, in their Ca^2+^‐binding characteristics. Specifically, organellar buffers exhibit a low affinity but high capacity for Ca^2+^ binding, enabling them to store large amounts of Ca^2+^. In contrast, cytosolic buffers have a high affinity but low capacity, allowing them to bind and release Ca^2+^ ions rapidly (Krebs et al. 2015).

Intracellular organelles such as the ER and mitochondria act as active Ca^2+^ stores, helping with Ca^2+^ buffering by sequestering and releasing Ca^2+^ when needed. This process is regulated through specialized Ca^2+^ pumps and channels in the membranes of these organelles, enabling Ca^2+^ flux between cellular compartments. Additionally, the plasma membrane indirectly supports Ca^2+^ buffering by removing Ca^2+^ from the cytosol through transporters like the plasma membrane Ca^2+^‐ATPase (PMCA) and the Na^+^/Ca^2+^ exchanger (NCX) (Fresu et al. 1999; Rose, Brian, et al. 2020).

**FIGURE 3:**
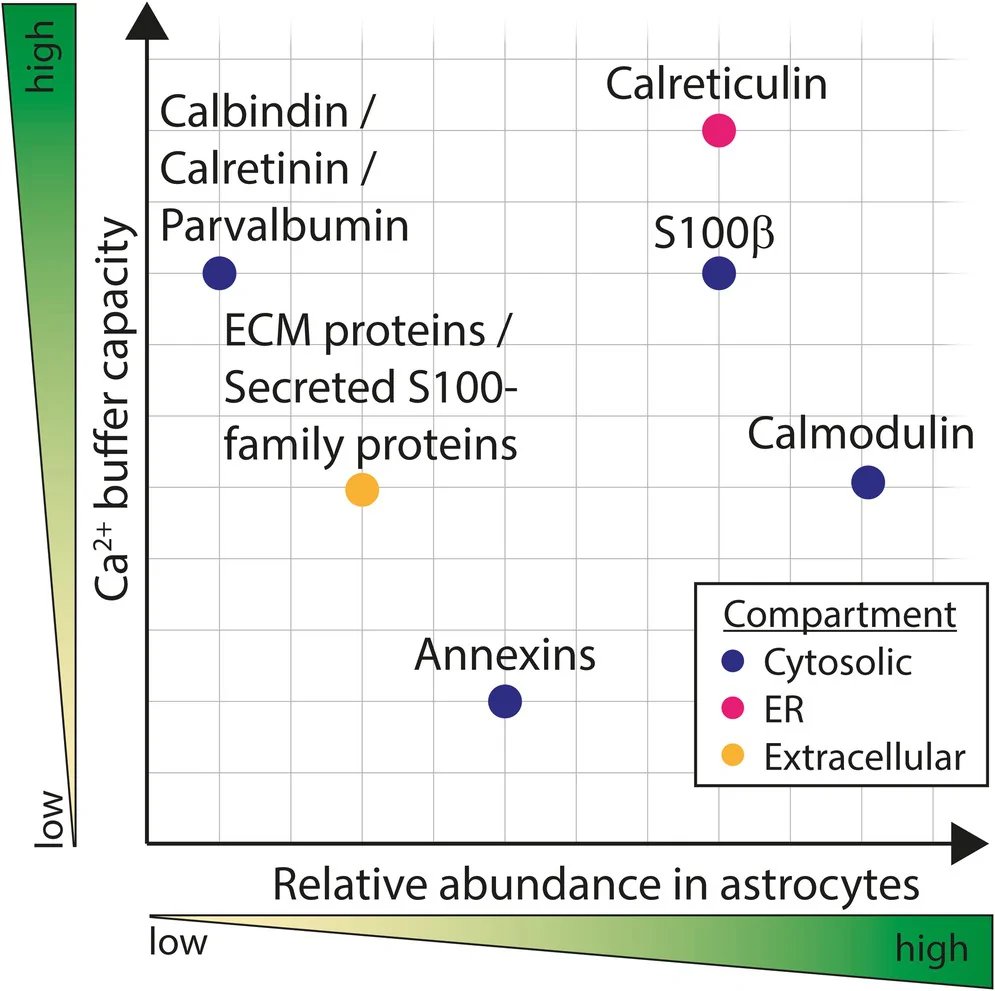
Relevance of Ca^2+^ buffers in astrocytes according to their abundance and buffer capacity. Astrocytic Ca^2+^ buffering relies on distinct compartment‐specific proteins. Calreticulin, localized in the ER lumen, provides high‐capacity, low‐affinity Ca^2+^ buffering that enables sustained Ca^2+^ storage and supports oscillatory Ca^2+^ release. In the cytosol, calmodulin and S100β are highly expressed and act as both moderate buffers and Ca^2+^ sensors that couple Ca^2+^ transients to downstream signaling pathways. Annexins are membrane‐associated and expressed at moderate levels in astrocytes, where they act as low‐affinity buffers. Calbindin, Calretinin, and Parvalbumin possess moderate to high Ca^2+^ buffer capacity but are of comparably low abundance in astrocytes. Extracellular matrix proteins and secreted proteins from the S100 family additionally bind Ca^2+^ in the extracellular space, shaping local Ca^2+^ availability for astrocytic processes. Collectively, these are the main buffering systems defining the characteristic slow, integrative, and spatially diffuse Ca^2+^ signaling in astrocytes. Abundance ranking is based on https://brain‐map.org/our‐research/cell‐types‐taxonomies/cell‐types‐database‐rna‐seq‐data, buffer capacity is described in the corresponding sections. ER, endoplasmic reticulum; ECM, extracellular matrix.

The relevance of a buffer for the Ca^2+^ activity in astrocytes is determined by its abundance and its buffering capacity (Figure 3). The most important endogenous buffers are described in the following:

Calreticulin is an ER‐associated chaperone and supposedly the most potent Ca^2+^ buffer in astrocytes. Its regulatory functions allow it to act as a gatekeeper for Ca^2+^, controlling ER Ca^2+^ supply, limiting access, and directing how Ca^2+^ is utilized (Michalak 2024). Calreticulin binds Ca^2+^ with high capacity (25 mol Ca^2+^ per mol of protein) and low affinity (*K*_d_ = 2 mM), buffering through its negatively charged amino acid residue‐enriched C‐domain (Nakamura et al. 2001). Even distal astrocytic elements like astrocyte endfeet at blood vessels contain ER structures (Boulay et al. 2017; Göbel et al. 2020; Soto and Khakh 2023), in which Calreticulin expression was shown to decrease with age, so age should be considered when modeling the impact of Calreticulin buffering (Zarate et al. 2021).

S100β is both abundant in astrocytes and a reasonably strong buffer (see Figure 3). It performs a dual role as a buffer and regulator of many physiological astrocyte functions. A notable feature of S100β and most other S100 proteins is that they often bind Ca^2+^ ions relatively weakly in the absence of a target protein, and upon binding to their target proteins, the Ca^2+^ binding affinity increases by approximately 200‐ to 400‐fold (Zimmer and Weber 2010).

Each S100β monomer binds Ca^2+^ through two hinge‐connected EF‐hand motifs: a carboxy‐terminal canonical EF‐hand motif and an amino‐terminal EF‐hand motif, which is unique among S100 proteins (Amburgey et al. 1995; Fritz and Heizmann 2006; Marenholz et al. 2004). Its secretion is triggered by intracellular Ca^2+^ mobilization, linking its activity to dynamic Ca^2+^ fluctuations in astrocytes (Leite et al. 2023). Overall, S100β functions as both a Ca^2+^ sensor and buffer, shaping rapid, localized Ca^2+^ signals essential for astrocyte physiology.

Calmodulin (CaM) is highly abundant in astrocytes but has moderate buffering capacity (Table 2). CaM controls astrocyte Ca^2+^ signaling by acting as a Ca^2+^ sensor that modulates the activity of Ca^2+^‐dependent proteins, such as Ca^2+^–CaM‐dependent protein kinase II (CaMKII) and calcineurin, and by affecting downstream intracellular processes like gliotransmitter release, gene transcription, and the coupling of Ca^2+^ to cyclic adenosine monophosphate (cAMP) synthesis via CaM‐sensitive adenylyl cyclases (ACs). Disruption of CaM‐dependent signaling pathways in astrocytes (e.g., inhibition of CaMKII) has been shown to cause abnormal Ca^2+^ oscillations, disrupted homeostasis, and changes in extracellular ATP release, emphasizing its vital role in shaping Ca^2+^ dynamics and feedback signaling. Overall, CaM allows astrocytes to translate Ca^2+^ signals into specific physiological responses necessary for their modulatory and homeostatic roles in the brain (Ashpole et al. 2013; Cooper et al. 1995; Willoughby and Cooper 2007; Yang and Tsai 2022).

**TABLE 2:** Endogenous Ca^2+^ buffers in astrocytes.

Name	Localization	Concentration	*K*_d_	Buffer capacity	Physiological function	Effect on Ca^2+^ signaling	References
Calreticulin	ER lumen	High, mM range	Low affinity (*K*_d_ ~200–600 μM)	Very high, each calreticulin molecule binds ~20–50 Ca^2+^ ions	High‐capacity ER Ca^2+^ buffer; regulates storage, and luminal Ca^2+^ dynamics, ER Ca^2+^ storage, protein folding, ER stress regulation	Acts as a Ca^2+^ reservoir, enabling the ER to store large amounts of Ca^2+^ without letting free luminal [Ca^2+^] rise excessively; prevents toxicity Enabling sustained oscillations and waves	Krebs et al. (2015), Michalak (2024)
S100β	Cytosol, nucleus, endfeet; secreted extracellularly, perisynaptic	μM range, enriched in astrocytes	Moderate affinity (*K*_d_ ~0.1–0.5 μM, 0.6 μM in endfeet)	Moderate (binds 2 Ca^2+^ ions/monomer)	Binds Ca^2+^ outside the cell; paracrine signaling (neurotrophic at low levels, neurotoxic at high)	Modulates cytosolic Ca^2+^ transients; secreted form influences extracellular Ca^2+^ buffering & paracrine signaling, S100β secretion is triggered by the increase in intracellular Ca^2+^, and indicates that this increase is due to Ca^2+^ mobilization from ER	Cristóvão et al. (2018), Leite et al. (2023), Santamaria‐Kisiel et al. (2006), Zimmer and Weber (2010)
Calmodulin (CaM)	Cytosol, subcellular membrane‐associated	2–10 μM	0.5–5 μM High affinity (*K*_d_ ~1–10 μM, site dependent)	Moderate‐low Each CaM molecule binds 4 Ca^2+^ ions	Moderate buffer and major Ca^2+^ sensor; activates CaMKs, calcineurin, AC; regulates transcription, metabolism, channels, volume regulation in astrocytes	Couples Ca^2+^ transients to signaling activity of Ca^2+^‐dependent proteins, such as CaMKII and calcineurin, disruption causes abnormal Ca^2+^ oscillations, altered homeostasis	Ashpole et al. (2013), Bender et al. (1992), Clapham (2007), Faas et al. (2011), Schwaller (2010), Sobue (2024)
Annexins (A1, A2, A7)	Cytosol, plasma membrane (Ca^2+^‐dependent binding)	μM range	Low affinity in solution (*K*_d_ mM), higher with membranes (~μM)	Variable	Ca^2+^‐ and phospholipid‐binding; regulate vesicle trafficking, endocytosis, exocytosis, and membrane repair	Contribute to slower, global Ca^2+^ waves by membrane recruitment and scaffolding	Bharadwaj et al. (2013), Rick et al. (2005), White et al. (2024)
Calbindin	Rare in astrocytes (specific subtypes/regions), reactive astrocytes	Very low	High affinity (*K*_d_ ~0.1–1 μM)	High per molecule	Classical fast Ca^2+^ buffers; when present, fine‐tune Ca^2+^ transients, predominantly in neurons	When present, accelerates decay of Ca^2+^ transients; minor role in astrocytes	Toyoshima et al. (1996)
ECM Proteins (proteoglycans, glycoproteins)	Extracellular matrix (perisynaptic, perivascular)	Not precisely quantified; locally high binding sites	Variable affinity for Ca^2+^	High extracellular capacity	Structural support, tissue homeostasis, signal transduction	Bind extracellular Ca^2+^, regulate diffusion & availability for astrocytic processes	Melrose (2024)

Abbreviations: AC, adenylyl cyclase; CaM, calmodulin; CaMKII, Ca^2+^–calmodulin‐dependent protein kinase II; CaMKs, Ca^2+^–calmodulin‐dependent protein kinases; ECM, extracellular matrix; ER, endoplasmic reticulum; *K*_d_, dissociation constant.

Annexins are a family of Ca^2+^‐ and phospholipid‐binding proteins expressed in astrocytes, with Annexin A1, A2, and A7 being the most prominent isoforms. Upon Ca^2+^ binding, they translocate to membranes where they scaffold and regulate exo‐ and endocytotic processes, thereby linking Ca^2+^ elevations to vesicular release and membrane repair. Additionally, their relatively slow, membrane‐dependent Ca^2+^ binding kinetics enable them to function as low‐affinity buffers and regulators of global Ca^2+^ waves, complementing the faster cytosolic EF‐hand proteins (Clemen et al. 2003; Patton et al. 2025; Shijo et al. 2019).

Calbindin is a classic EF‐hand Ca^2+^‐binding protein abundantly expressed in neurons, but its expression in astrocytes is low or absent under physiological conditions. Some studies have reported expression in reactive astrocytes, where it may serve as a high‐affinity, fast Ca^2+^ buffer to accelerate the decay of Ca^2+^ transients. However, in mature cortical and hippocampal astrocytes, calbindin is not considered a major contributor to Ca^2+^ buffering compared to proteins like S100β or calreticulin (Baimbridge et al. 1992; Mattson et al. 1995; Wernyj et al. 1999).

Similarly, parvalbumin is a high‐affinity, slow‐onset EF‐hand Ca^2+^‐binding protein that functions as a mobile intracellular Ca^2+^ buffer but was only shown to be expressed by a subset of reactive astrocytes (Lichvarova et al. 2019; Szabolcsi and Celio 2014). Therefore, parvalbumin is considered subordinate for astrocyte Ca^2+^ buffering.

Extracellular matrix (ECM) proteins, especially proteoglycans and glycoproteins, influence astrocytic Ca^2+^ signaling by binding divalent cations like Ca^2+^ through negatively charged sulfate and carboxyl groups, thereby regulating the local availability of free extracellular Ca^2+^. This extracellular buffering affects the driving force for Ca^2+^ influx via astrocytic channels and transporters (e.g., transient receptor potential (TRP), purinergic receptors (P2X), Ca^2+^ release‐activated Ca^2+^ modulator 1 (Orai1), see below) and also controls the diffusion of Ca^2+^ and gliotransmitters in perisynaptic and perivascular microdomains. In pathological conditions such as injury or gliosis, ECM remodeling changes Ca^2+^ buffering capacity, which can profoundly affect astrocyte excitability and intercellular Ca^2+^ wave propagation (Egelman and Montague 1999; Mattson et al. 1995; Newman et al. 1995).

### 2.3 Ca^2+^ Mobility‐Channels and Transporters

Organelles relevant to Ca^2+^ buffering include mainly the ER and mitochondria. Although lysosomes participate in Ca^2+^ homeostasis, their contribution to bulk Ca^2+^ buffering can be regarded as minor due to their small storage volume. Therefore, lysosomes are not further discussed in this review (for an overview see Chen, Yejun, et al. 2025). The transport ways of Ca^2+^ need to cross the membranes of these organelles as well as the outer plasma membrane.

A balance between influx and extrusion pathways regulates astrocytic plasma membrane Ca^2+^ homeostasis. Channels responsible for these fluxes can be characterized by their activation mechanism. Ionotropic Purinergic P2X/P2Y receptors are ligand‐gated ion channels responding to ATP, shaping rapid, localized cytosolic Ca^2+^ transients in astrocytes (Illes et al. 2012; Fischer et al. 2009). TRP channels are a large family of polymodal cation channels expressed in astrocytes that function as non‐selective Ca^2+^‐permeable channels and contribute to receptor‐ and store‐operated Ca^2+^ entry (SOCE) as well as microdomain Ca^2+^ transients (Malarkey et al. 2008; Shigetomi et al. 2012; Skerjanz et al. 2024; Verkhratsky et al. 2013). SOCE, controlled by the Orai1 channel and ER Ca^2+^ sensor stromal interaction molecule 1 (STIM1), provides sustained Ca^2+^ influx after ER‐store depletion, thereby supporting oscillatory signaling and intercellular Ca^2+^ waves. For Ca^2+^ clearance, the PMCA ensures high‐affinity, ATP‐driven extrusion. At the same time, NCX contributes to low‐affinity, high‐capacity Ca^2+^ removal and can operate in reverse mode under altered conditions, that is, upon membrane depolarization or Na^+^ accumulation in the cytosol, allowing Ca^2+^ entry during astrocytic activity (Kwon et al. 2017; Pham et al. 2021; Rose, Ziemens, and Verkhratsky 2020; Shigetomi et al. 2016).

Astrocytes further express all three classes of ionotropic glutamate receptors (iGluRs), namely α‐amino‐3‐hydroxy‐5‐methyl‐4‐isoxazolepropionic acid receptors (AMPARs), kainic acid receptors (KARs), and N‐methyl‐D‐aspartate receptors (NMDARs), which can contribute to intracellular Ca^2+^ elevation either directly or via permitting Na^+^ influx, which in turn drives the NCX in reverse mode to cause Ca^2+^ influx (Cuellar‐Santoyo et al. 2023; Hadzic et al. 2016; Smith et al. 2000; Ziemens et al. 2019). Although not electrically excitable, astrocytes express several voltage‐gated Ca^2+^ channels (VGCCs) which can be activated by depolarization secondary to neurotransmitter uptake or ion fluxes and contribute modest Ca^2+^ influx that supplements intracellular store release (Denaroso et al. 2023).

The sarco/ER Ca^2+^‐ATPase (SERCA) actively transports Ca^2+^ from the cytosol back into the ER lumen, refilling ER Ca^2+^ stores and maintaining the reservoir required for oscillatory Ca^2+^ release through inositol trisphosphate (IP_3_) receptors (IP_3_Rs). By tightly regulating ER Ca^2+^ content, SERCA supports repetitive Ca^2+^ transients and intercellular Ca^2+^ waves and functions as an indirect cytosolic Ca^2+^ buffer, accelerating the clearance of Ca^2+^ after signaling events. Inhibition of SERCA (e.g., by thapsigargin) depletes ER stores, abolishes IP_3_‐mediated oscillations, and profoundly alters astrocytic Ca^2+^ dynamics (Lo et al. 2002; Morita and Kudo 2010; Simpson and Russell 1997; Verkhratsky and Nedergaard 2018).

A major source of cytosolic Ca^2+^ elevations in astrocytes is the release from the ER, which occurs primarily through two pathways: IP_3_ receptor activation and Ca^2+^‐induced Ca^2+^ release mediated by Ca^2+^‐sensitive ryanodine receptors (RyRs) (Furuichi et al. 1994; Lalo and Pankratov 2024; Matyash et al. 2002). While many studies focus on the IP_3_ axis, especially IP_3_R 2 (Denizot et al. 2019; Fernández de la Puebla et al. 2025; Monai and Hirase 2016; Wang et al. 2021), in recent years, RyRs in astrocytes have gained much attention and are increasingly considered relevant for astrocyte function (Lalo and Pankratov 2024; Skowrońska et al. 2020).

Mitochondrial Ca^2+^ uptake in astrocytes is primarily mediated by the mitochondrial Ca^2+^ uniporter (MCU), a low‐affinity, high‐capacity channel that allows rapid Ca^2+^ entry into the matrix during cytosolic Ca^2+^ rises, particularly in microdomains near the ER and plasma membrane. Ca^2+^ rise in mitochondria activates dehydrogenases and stimulates the tricarboxylic acid cycle and ATP production (Chen, Ye, et al. 2025; Tarasov et al. 2012). The accumulated Ca^2+^ is extruded via the mitochondrial Na^+^/Ca^2+^ exchanger (NCLX), which maintains mitochondrial Ca^2+^ homeostasis and prevents Ca^2+^ overload. Together, MCU and NCLX dynamically regulate mitochondrial Ca^2+^, thereby coupling astrocytic Ca^2+^ signaling to ATP production and protecting cells from Ca^2+^‐induced stress (Parnis et al. 2013; Rizzuto et al. 2012).

Last but not least, Ca^2+^ waves can spread from one astrocyte to another within the gap‐junction‐coupled astrocyte syncytium (intercellular Ca^2+^ events), posing the need to account for cellular connectivity in computational modeling (Giaume and Venance 1998; Leybaert and Sanderson 2012).

## 3 Artificial Ca^2+^ Buffering Through Extrinsic Factors

### 3.1 Ca^2+^ Indicators and Sensors

Ca^2+^ indicators and sensors operate by binding free Ca^2+^ ions and turning this interaction into a measurable fluorescence signal. This allows for real‐time monitoring of intracellular Ca^2+^ levels and transients within living cells, such as astrocytes. These sensors include synthetic chemical indicators, such as small fluorescent molecules like Fura‐2 or Oregon Green BAPTA‐1, which are known for their high sensitivity and rapid kinetics but require invasive loading and potentially perturb cellular Ca^2+^ buffering. In contrast, GECIs, like GCaMPs, use protein‐based sensors with Ca^2+^‐binding domains—most often CaM—fused to fluorescent proteins. When Ca^2+^ binds, it causes conformational changes that increase fluorescence, enabling targeted, long‐term monitoring in genetically specified cells, although they often have slower kinetics compared to synthetic dyes (Zeug et al. 2024).

The principles of Ca^2+^ affinity, binding kinetics (*k*_on_, *k*_off_), and buffering power have already been explained above. However, for biosensor readout, the concept of the half‐maximal effective concentration (EC_50_) is crucial for interpreting sensor outputs, even though it is often less clearly defined in astrocyte research. The *K*_d_ of a sensor indicates its intrinsic biochemical affinity, representing the free Ca^2+^ concentration at which half of the sensor's binding sites are occupied. In contrast, EC_50_ is derived from empirical dose–response analyses and specifies the Ca^2+^ concentration that produces half‐maximal sensor fluorescence or signal output. For Ca^2+^ indicators, *K*_d_ assumes the indicator concentration to be much smaller than the total Ca^2+^ concentration (tracer condition). When indicator concentration becomes comparable to *K*_d_, Ca^2+^ depletion shifts to higher values via the quadratic binding equation (Buchwald 2025; Hulme and Trevethick 2010; Keener and Sneyd 2025). Unlike *K*_d_, EC_50_ incorporates the complexities of multisite cooperativity, fluorescence transduction efficiency, and conformational dynamics that modulate sensor performance.

Proteins like CaM, which are essential components of GCaMP sensors, have four EF‐hand Ca^2+^ binding sites that enable cooperative Ca^2+^ binding. Ca^2+^ binding induces a conformational change in CaM that wraps around the adjacent M13 peptide (derived from myosin light‐chain kinase), which in turn closes interfaces within the circularly permuted GFP domain to enhance fluorescence (Nagai et al. 2001). Point mutations can thereby tune the binding kinetics such that specific fast (GCaMP6f) or slow (GCaMP6s) variants can be derived (T.‐W. Chen et al. 2013). Troponin C, used in TnXXL sensors, likewise features four EF‐hand Ca^2+^ binding sites (two low‐affinity sites in the N‐lobe and two high‐affinity sites in the C‐lobe of the engineered chicken fast skeletal muscle TnC variant). Upon Ca^2+^ binding—primarily to the N‐lobe sites—TnC undergoes a conformational change that brings the attached CFP and YPet fluorophores into closer proximity (or alters their orientation), increasing fluorescence resonance energy transfer (FRET) between them (Geiger et al. 2012; Mank et al. 2008). The presence of multiple binding sites and their cooperative interactions significantly influence the observed EC_50_ and the sensor's dynamic range. Typically, the response of these multisite Ca^2+^ biosensors to rising Ca^2+^ concentration can be described by a sigmoidal function. This sigmoidal response is mathematically modeled by the Hill equation, where the Hill coefficient indicates both the steepness of the response curve and the degree of cooperativity among binding sites. In proteins like CaM or troponin C, a Hill coefficient greater than one indicates positive cooperativity—meaning binding of one Ca^2+^ ion increases the likelihood of subsequent ions binding. This results in an ultrasensitive, switch‐like sensor response. With positive cooperativity, the multiple binding sites produce a sharper sigmoidal transition between low and high sensor activity states, rather than a gradual response. This is vital for accurately detecting physiological Ca^2+^ signals. These features have been extensively studied in Ca^2+^ sensors and are especially important for understanding astrocytic Ca^2+^ signals, where cooperative mechanisms enhance the sensitivity and specificity to complex, spatially and temporally variable Ca^2+^ transients (Mank et al. 2008; Nakai et al. 2001; Roelse et al. 2019; Sun et al. 2013; Zhang et al. 2023).

Moreover, Ca^2+^ indicators utilize several optical modalities for readout. Fluorescence intensity is the most common, with relative changes obtained as the increase in brightness of GCaMP when Ca^2+^ binds. Alternatively, fluorescence lifetime measurements determine how long a fluorophore remains excited before emitting a photon, providing a signal less affected by sensor concentration or lighting conditions. Less commonly used are fluorescence polarization or anisotropy, which reflect changes in molecular rotation or conformational state upon Ca^2+^ binding, providing additional layers of functional information. Each readout modality can display a different EC_50_. For instance, an indicator showing changes in both fluorescence intensity and lifetime upon Ca^2+^ binding can have distinct EC_50_ values for intensity versus lifetime measurements, as observed for jRCaMP1h (personal observations). These differences influence the interpretation and quantitative reliability of Ca^2+^ imaging, with implications for precisely analyzing astrocyte Ca^2+^ signaling (Pérez Koldenkova and Nagai 2013; Ross et al. 2018).

While *K*_d_ conveys fundamental binding and buffering properties of Ca^2+^ sensors, EC_50_ synthesizes these molecular interactions with the sensor's output characteristics. Understanding both parameters, along with the multisite binding nature of CaM and troponin, and the selected fluorescence readout modality, is essential for accurately probing Ca^2+^ dynamics in astrocytes.

### 3.2 Ca^2+^ Indicator Dynamics

While the EC_50_ parameter offers a useful measure of sensor sensitivity, it does not describe the kinetic aspects of Ca^2+^ binding and release. Unlike the well‐established kinetic constants *k*_on_ and *k*_off_, which detail the rates of Ca^2+^ association and dissociation—essential for understanding sensor response times—there is no direct kinetic equivalent to EC_50_, such as a hypothetical *EC*_on_ or *EC*_off_ (Hoare et al. 2020).

In practice, researchers often characterize the kinetics of the Ca^2+^ indicator by analyzing its response to specific stimuli under controlled conditions. For example, they may assess Ca^2+^ transients triggered by a set number of neuronal action potentials (e.g., 1AP, 10APs, 100APs). These stimulus‐evoked responses provide indirect insight into the temporal dynamics of sensor signals and, consequently, Ca^2+^ kinetics. Such methods have been quite successful in neuronal studies where action potentials produce well‐defined, repeatable Ca^2+^ influx events (T.‐W. Chen et al. 2013).

However, this method is much less effective in astrocyte research, where responses are usually not triggered by action potentials but by complex, often locally confined, and less precisely timed Ca^2+^ signals. Astrocytes exhibit spontaneous and heterogeneous Ca^2+^ activity that is influenced by a wide range of stimuli, including neurotransmitters, neuromodulators, and intrinsic cellular mechanisms, making direct kinetic characterization more challenging. Thus, astrocyte researchers must rely on alternative approaches such as computational modeling of Ca^2+^ dynamics or examining Ca^2+^ transient kinetics in response to drugs or spontaneous activity, while understanding the limitations and complexities of these approaches.

Consequently, while the equilibrium parameter EC_50_ reliably gauges sensor sensitivity, a comprehensive kinetic understanding often requires multifaceted approaches that combine biochemical measurements of k_on_ and k_off_, sensor response profiling under physiological or synthetic stimulations, and computational modeling. These challenges underline the need for refined kinetic descriptors and standardized protocols tailored to the intricate temporal and spatial Ca^2+^ signaling landscapes unique to astrocytes.

### 3.3 Ca^2+^ Indicator Buffering

The introduction of Ca^2+^ biosensors into astrocytes can significantly influence the native Ca^2+^ dynamics they aim to report, and this impact is intricately tied to the relationship between the sensor's intrinsic Ca^2+^ affinity, *K*_d_, and its empirical EC_50_. This distinction can lead to scenarios in which the *K*_d_ of a Ca^2+^ indicator is significantly lower than its EC_50_, or vice versa, which in turn impacts how the indicator modifies intracellular Ca^2+^ dynamics.

When a sensor's *K*_d_ is much lower than its EC_50_, it indicates a higher chemical affinity than what fluorescence detection suggests, resulting in potent Ca^2+^ binding that markedly augments the cell's buffering capacity (Buchwald 2025). This can dampen the amplitude of Ca^2+^ transients in astrocytes and slow Ca^2+^ clearance, potentially leading to prolonged and attenuated Ca^2+^ signals that do not faithfully represent native physiology. Examples of such discrepancies have been reported, in which high‐affinity indicators inadvertently sequester Ca^2+^ more strongly than anticipated based on their fluorescence response, thus influencing the apparent Ca^2+^ kinetics. Conversely, indicators with a *K*_d_ higher than their EC_50_ may exhibit weaker intrinsic buffering despite exhibiting fluorescence changes at lower Ca^2+^ concentrations. These sensors may minimally perturb intracellular Ca^2+^ dynamics, preserving physiological amplitudes and kinetics, but possibly at the cost of sensitivity or dynamic range (Buchwald 2025; Ribeiro‐Do‐Valle et al. 1994; Robinson et al. 2023). Figure 2B schematically separates the biochemical affinity from the optical sensitivity of Ca^2+^ indicators, illustrating how different combinations of *K*_d_ and EC_50_ distort both the underlying Ca^2+^ transient and its reported fluorescence.

The morphological complexity of astrocytes further complicates how exogenous buffering influences Ca^2+^ transients. In fine perisynaptic processes—small‐volume compartments with highly localized and rapid Ca^2+^ signals—the presence of even modest Ca^2+^ buffering capacities introduced by indicators can dramatically shape the amplitude and duration of these transients by sequestering free Ca^2+^ and slowing diffusion. For instance, the membrane‐targeted GCaMP variants reveal microdomain Ca^2+^ signals in astrocyte processes that were previously undetectable with cytosolic indicators, emphasizing the role of the spatial distribution of buffering capacity. In contrast, larger compartments such as the soma or main branches possess greater volume and buffering heterogeneity, which can mitigate but not eliminate the perturbations induced by exogenous sensor buffering. Indeed, the local concentration and distribution of Ca^2+^ indicators can mask or distort Ca^2+^ transients, leading to erroneous interpretation of Ca^2+^ kinetics, such as underestimation of peak amplitudes or mischaracterization of decay phases (Denizot et al. 2019; Shigetomi et al. 2010).

Such perturbations underscore the importance of carefully selecting Ca^2+^ indicators whose biochemical and optical properties most closely match the experimental conditions in astrocyte research. A comprehensive overview of available indicators and their applications is provided elsewhere (Lohr et al. 2021; Zeug et al. 2024). Understanding the subtle differences between *K*_d_ and EC_50_, as well as their impact on sensor buffering capacity, improves the interpretation of Ca^2+^ imaging data and prevents misleading conclusions about astrocyte Ca^2+^ dynamics. This knowledge also aids in developing sensors that enable the minimally invasive monitoring of the complex spatiotemporal Ca^2+^ movements that are typical of astrocytes.

## 4 The Implementation of Ca^2+^ Buffering Mechanisms in Computational Modeling

Computational biophysical models can offer mechanistic insights into complex biological systems (Keener and Sneyd 2025). By representing biological systems–such as astrocytes–through mathematical equations, these models allow scientists to simulate and analyze the underlying mechanisms of molecular, cellular, and network interactions, helping to understand how different components of a system collaborate. Ca^2+^ buffering in astrocytes has been modeled both implicitly, that is, by using effective rate constants and diffusion coefficients, and explicitly, that is, by including separate equations for Ca^2+^ buffers. The implicit approach captures the effects of fast Ca^2+^ buffering through relevant parameters (Bezerra and Roque 2024; Denizot et al. 2019; Freund et al. 2024; Gordleeva et al. 2019; Höfer et al. 2001; Kenny et al. 2018; Moshkforoush et al. 2021; Oschmann et al. 2017; Thapaliya et al. 2023; Ullah et al. 2006; Verisokin et al. 2021; Vuillaume et al. 2021; Xu et al. 2026), while the explicit approach directly models the binding and unbinding of Ca^2+^ to buffer molecules (Denizot et al. 2019; Hadfield et al. 2013; Jha et al. 2020; López‐Caamal et al. 2014; Savtchenko et al. 2018; Shoemaker and Bekkouche 2025; Skupin et al. 2010).

Notably, in endogenous Ca^2+^ buffer models, the cytosolic buffering alone is often modeled since the buffering effect in the ER is relatively small compared to that in the cytosol. Therefore, buffering in the ER is often neglected in many models (Hadfield et al. 2013; López‐Caamal et al. 2014).

Below, we summarize key computational models with Ca^2+^ buffering in astrocytes, focusing on recent publications in the last decade (after 2017) and organized by publication year. Thus, computational models with Ca^2+^ buffering, as covered in the review by Manninen et al. (2018), have not been considered here. All models calculate cytosolic buffering based on the law of mass action kinetics. Table 3 provides the buffer type, the part of the cell modeled, and the equations describing the buffer kinetics and cytosolic Ca^2+^ concentration over time.

**TABLE 3:** Summary of computational astrocyte models that include Ca^2+^ buffering.

First author et al. (year)	Ca^2+^ buffers (endogenous and exogenous) and kinetics type	ER stores	Mitochondria	Morphology/spatial detail	Key features
Kenny et al. (2018)	Implicit buffering As buffering factor: Rapid‐buffering factor for endogenous and exogenous buffer, Ca^2+^ buffering rate at endfoot and soma	IP_3_Rs; SERCA, Ca^2+^ leak	Not included	Simplified multicompartmental model of the neurovascular unit (NVU)	Extends a NVU model to include glutamate‐driven astrocytic Ca^2+^ signaling, EET production, and stretch‐activated TRPV4 Ca^2+^ influx at endfeet
Savtchenko et al. (2018)	Explicit: Endogenous and exogenous Separate variables for free Ca^2+^ and Ca^2+^ buffer complexes with binding/unbinding	IP_3_Rs; SERCA	Not included	3D detailed multicompartmental cell model	3D compartmental morphology, NEURON integration, glutamate and K^+^ signaling, multi‐scale modeling
Denizot et al. (2019, 2022)	Explicit: Endogenous and exogenous for comparison with implicit buffering model Separate variables for free Ca^2+^ and Ca–buffer complexes with binding/unbinding	ER release/uptake with partial coverage	Not included	Stylized “nodes‐and‐shafts” matched to EM; realistic morphologies	Microdomain framework, morphology–function relationship
Jha et al. (2020)	Explicit: Immobile endogenous buffering Deterministic reaction–diffusion	Not included	Not included	3D finite‐element astrocyte geometry	3D morphology, voltage gated Ca^2+^ channel (VGCC) signaling
Moshkforoush et al. (2021)	Implicit buffering As buffering factor	IP_3_Rs; SERCA, Ca^2+^ leak	Not included	Spatially lumped astrocyte compartment	Stochastic optogenetic (ChR2) model of astrocyte Ca^2+^ dynamics
Verisokin et al. (2021)	Implicit buffering Lumped with Ca^2+^ concentration	IP_3_Rs; SERCA, Ca^2+^ leak	Not included	Data‐driven spatial 2D templates	Realistic astrocyte morphology, spatially structured astrocyte network, multiscale Ca^2+^ dynamics
Vuillaume et al. (2021)	Implicit endogenous buffering As buffering factor	IP_3_Rs; SERCA, Ca^2+^ leak	Not included	Astrocytes modeled as non‐spatial units arranged in a layered network	Tripartite synapse/network model including gliotransmission
Thapaliya et al. (2023)	Implicit: As in Kenny et al. (2018)	IP_3_Rs; SERCA, Ca^2+^ leak	Not included	Simplified two‐compartmental cell model	Model fitted to cortical vs. hippocampal Na^+^ and Ca^2+^ data
Freund et al. (2024)	Implicit buffering Lumped with Ca^2+^ concentration	IP_3_Rs; SERCA, Ca^2+^ leak	Not included	3D detailed multicompartmental cell model	Astrocyte morphological heterogeneity, 3D compartmental morphology, morphology–function relationship, glutamate, K^+^, and Na^+^ signaling
Bezerra and Roque (2024)	Implicit buffering Lumped with Ca^2+^ concentration	IP_3_Rs; SERCA, Ca^2+^ leak	Not included	Simplified multicompartmental cell model	Compartmental morphology, glutamate and dopamine signaling
Shoemaker and Bekkouche (2025)	Explicit: Endogenous buffering (Calbindin) Mass‐action reversible binding kinetics between free Ca^2+^ and an immobile buffer (Calbindin), reaction–diffusion for bound‐buffer species	IP_3_Rs; SERCA, Ca^2+^ leak, RyR channels	Not included	Idealized 2D/3D geometries of dendrite‐like processes and discoid cell body (associated with an astrocyte)	Analyzes how morphology, receptor density, diffusion, and buffering shape wave speed, amplitude, and stability
Xu et al. (2026)	Implicit endogenous buffering As buffering factor	IP_3_Rs; SERCA, Ca^2+^ leak	Not included	Single‐compartment astrocyte model plus neuron and arteriole	Neurovascular coupling (NVC) in epilepsy, explores firing‐pattern bifurcations and NVC changes under ischemia, hypoxia, and astrocyte dysfunction

Abbreviations: ChR2, Channelrhodopsin‐2; EET, epoxyeicosatrienoic acid; EM, electron microscopy; ER, endoplasmic reticulum; IP_3_Rs, IP_3_ receptors; NVC, neurovascular coupling; NVU, neurovascular unit; ODE, ordinary differential equation; RyR, ryanodine receptors; SERCA, sarco/ER Ca^*2*+^‐ATPase; TRPV4, transient Receptor Potential Vanilloid 4; VGCC, voltage‐gated Ca^*2*+^ channel.

The four recent models that explicitly describe Ca^2+^ buffering are those of Denizot et al. (2019), Jha et al. (2020), Savtchenko et al. (2018), and Shoemaker and Bekkouche (2025), which use either a dedicated pool of mobile buffer or validate implicit reductions by comparing them with explicit multi‐buffer systems. Savtchenko et al. (2018) describe a detailed multicompartmental cell model called ASTRO, based on 3D reconstructions of hippocampal astrocytes, in which the ‘stem tree’ is obtained from optical images and the nanoscopic structure from 3D electron microscopy (EM) images. Their main findings indicate that astrocyte structure significantly influences intracellular signaling, potassium and glutamate uptake, and Ca^2+^ dynamics, including waves and microdomain signals. The Ca^2+^ buffering equation used in the ASTRO framework is the same as in the NEURON framework (Hines and Carnevale 1997). It is based on classic reaction kinetics between free Ca^2+^ and a buffer molecule, forming a bound complex. All endogenous buffering is modeled with a single mobile buffer pool. This explicit modeling demonstrates how the properties of mobile Ca^2+^ buffers affect the amplitude, speed, and spatial spread of astrocytic Ca^2+^ waves. Denizot et al. (2019) present a spatially explicit, particle‐based computational model of IP_3_R‐mediated Ca^2+^ signaling in fine astrocytic processes. They mainly describe Ca^2+^ buffering implicitly by reducing the diffusion coefficient for Ca^2+^. However, to confirm that their implicit model accounts for buffer effects, they added exogenous buffer molecules, specifically GCaMP6s and GCaMP6f, along with endogenous Ca^2+^ buffers such as slow and fast calbindin and parvalbumin. Contrary to simulation results without explicit buffers, simulations including both exogenous and endogenous buffers produced similar results and more accurately matched experimental data. GCaMP6 appears to alter local Ca^2+^ concentration and increases the duration of Ca^2+^ peaks. When comparing GCaMP6s and GCaMP6f signals, the latter show a higher amplitude and shorter duration than GCaMP6s signals. Increasing the GCaMP6 concentration results in a longer peak duration but does not change the peak frequency of spontaneous Ca^2+^ signals.

Jha et al. (2020) developed a 3D finite‐element reaction–diffusion model of Ca^2+^ dynamics in an astrocyte with a voltage‐gated Ca^2+^ channel at the membrane. They include explicit immobile buffering (separate free Ca^2+^–buffer equations) and investigate how astrocyte geometry and buffer properties shape spatial cytosolic Ca^2+^ distributions during voltage‐gated Ca^2+^ channel activation. Shoemaker and Bekkouche (2025) present a mechanistic reaction–diffusion model of traveling Ca^2+^ waves in idealized cellular structures (dendrite‐like or in general cellular processes and a discoid cell body, such as from an astrocyte) that includes IP_3_ receptors, ryanodine receptors, SERCA, and plasma membrane pumps, diffusion, and explicit cytosolic buffering. They find that wave speed and amplitude are strongly sensitive to ER Ca^2+^ load, receptor densities, cytosolic diffusion, and Ca^2+^ buffering, and that even downgraded ryanodine receptor conductance can markedly enhance wave propagation.

The degree of anatomical realism is another key factor distinguishing these frameworks. Savtchenko et al. (2018) and Freund et al. (2024) use detailed 3D reconstructions or multi‐compartment models that closely match the actual morphology of astrocytes, emphasizing the significant effects of spatial compartmentalization on microdomain signaling and inter‐compartment communication. Denizot et al. (2019) take it a step further by using stylized shapes aligned with EM‐reconstructed geometries, highlighting the strong influence of structural “nodes and shafts” on Ca^2+^ kinetics. In contrast, Bezerra and Roque (2024), Jha et al. (2020), and Shoemaker and Bekkouche (2025) offer a simplified yet morphologically organized cell model, while Verisokin et al. (2021) find a middle ground with data‐driven 2D spatial templates for building larger networks.

Another essential part is the handling of intracellular Ca^2+^ stores—especially the ER—and the function of mitochondrial buffering. The most recent models now include ER dynamics with varying complexity, incorporating IP_3_R cluster‐mediated release, SERCA pumps, and leak currents; however, explicit modeling of mitochondrial buffering remains rare or is often omitted. This simplification can be justified in specific signaling scenarios, but it limits the understanding of situations in which mitochondrial sequestration or release would significantly influence Ca^2+^ wave propagation and recovery times.

Kinetics are typically deterministic and governed by nonlinear differential equations, but some models—especially those addressing noise‐driven cellular processes or aiming to replicate stochastic channel gating—incorporate stochastic reaction–diffusion formalisms. Savtchenko et al. (2018), for example, employ a stochastic approach within a multi‐scale NEURON‐driven environment, enabling a faithful representation of random channel activity. In contrast, models such as Freund et al. (2024), Kenny et al. (2018), and Bezerra and Roque (2024) maintain a deterministic framework. Denizot et al. (2019) notably combine both approaches, using deterministic and stochastic methods to explore the connection between geometry and Ca^2+^ microdomain dynamics. In the extremely small volumes of fine astrocytic processes, molecular copy numbers are so low that stochastic noise becomes a dominant feature of Ca^2+^ dynamics. Increased Ca^2+^ buffering (including by indicators like GCaMP) in those narrow spaces decreases peak probability and amplitude while increasing peak duration (Denizot et al. 2022).

Mathematically, the form of the buffering equations is essentially the same across all sources that use explicit buffer modeling, which is basically another way of writing equation (1):

(3) $$\begin{array}{c} \frac{d\left[{\mathrm{Ca}}^{2+}\right]}{\mathrm{dt}}=-k_{\mathrm{on}}\left[B\right]\left[{\mathrm{Ca}}^{2+}\right]+k_{\mathrm{off}}\left[\mathrm{CaB}\right], \end{array}$$

where $\left[{\mathrm{Ca}}^{2+}\right]$ denotes the concentration of free Ca^2+^ ions and $\left[\mathrm{CaB}\right]$ the concentration of Ca^2+^ bound to the buffer. Additionally, possible spatial diffusion and source/flux terms were incorporated into the equations of the models mentioned above (see also Table 3). Savtchenko et al. (2018), Jha et al. (2020), and Denizot et al. (2019) offer the most flexible, biophysically complete approaches, allowing for multiple buffers (mobile and immobile), each with its own parameters and diffusion coefficients.

In summary, recent progress shows a dynamic modeling field where spatial and organizational complexity, the level of buffering detail, and the inclusion of stochasticity are tailored to the specific biological question. Models with advanced spatial templates and explicit buffer kinetics excel at examining microdomain and geometry‐effect relationships but require significant computational resources. On the other hand, simplified or lumped buffer models allow for broader studies of network and population‐level phenomena, though they may sometimes overlook important physiological mechanisms and heterogeneity. This variation in approach reflects the biological complexity of astrocyte Ca^2+^ handling itself and highlights the need for ongoing methodological diversity and comparison across different scales and levels of detail.

## 5 Discussion and Final Summary

Astrocytes detect synaptic activity via neurotransmitter‐induced Ca^2+^ elevations, voltage‐gated channels, and extracellular ion alterations. Their internal buffering capacity modulates the amplitude, duration, and propagation of these Ca^2+^ signals, which regulate the release of gliotransmitters (such as glutamate, ATP, and D‐serine). This release, in turn, fine‐tunes synaptic transmission and plasticity (Khakh and McCarthy 2015; Sasaki et al. 2014; Semyanov et al. 2020).

This review underscores the profound impact of Ca^2+^ buffering on astrocytic physiology, which is reflected in the interpretation of data and the development and application of computational models. The intricate interplay between various endogenous and exogenous buffers shapes the amplitude, kinetics, and spatial properties of astrocyte Ca^2+^ signals, which serve as the backbone for many of the cell's homeostatic and signaling functions. Our synthesis highlights that both immobile and mobile buffers—including proteins such as S100β, calreticulin, CaM, and annexins, as well as organelles like the ER and mitochondria—act in compartment‐specific ways to define unique signaling microdomains and the propagation of Ca^2+^ waves within the astrocyte syncytium.

One critical aspect emerging from recent experimental and computational modeling work is the diversity and plasticity of endogenous buffering mechanisms. Different astrocytic compartments (soma, branches, and fine processes) possess distinct compositions and concentrations of buffer proteins, which dynamically modulate local signaling in both health and disease. The interplay between buffer kinetics (*k*_on_, *k*_off_), binding affinities, and local concentrations determines not only the speed and intensity of Ca^2+^ transients but also their capacity for integration and impact on downstream cascades, such as metabolic regulation, gene transcription, and gliotransmitter release. Our review emphasizes the importance of considering ions such as Mg^2+^ and H^+^, which compete with Ca^2+^ for binding sites and further modulate the effective buffering power in vivo—an aspect often neglected in both experimental interpretations and computational simulations. We noticed inconsistent use of the terms endogenous and its subtypes, immobile and mobile buffering, in some of the cited papers, which makes information collection and comparison challenging.

The introduction of exogenous buffers, including synthetic chelators, fluorescent dyes, and genetically encoded Ca^2+^ indicators (GECIs), presents both opportunities and challenges. While invaluable for cellular imaging, these buffers can distort the native Ca^2+^ concentrations, slowing transients and altering signal amplitude, especially in fine astrocytic processes with limited volume (Wang et al. 1997). Consequently, this has effects on neuro‐ and gliotransmission (Sibille et al. 2015). The distinction between a sensor's chemical affinity (*K*_d_), empirical sensitivity (EC_50_), and the resulting buffering effect is essential yet often understudied. Our analysis recommends meticulous calibration and kinetic characterization of indicators and advocates for simulation frameworks that explicitly account for sensor‐induced buffering when interpreting imaging data.

Several open questions and controversies persist in the field (Khakh and McCarthy 2015; Semyanov et al. 2020). One ongoing debate is the physiological significance of spontaneous Ca^2+^ signals in astrocytes—whether these represent meaningful regulatory events or simply stochastic cellular noise. In addition, the types, locations, and regulatory mechanisms of endogenous Ca^2+^ buffers in astrocytes remain incompletely understood, with evidence suggesting heterogeneous buffer properties across subpopulations and subcellular compartments. This diversity poses a particular challenge for accurate experimental mapping and model development. The clinical relevance of altered astrocytic Ca^2+^ buffering—for diseases such as neurodegeneration, epilepsy, or psychiatric conditions—remains both speculative and underexplored, largely due to a dearth of direct human data. More broadly, the precise mechanisms by which Ca^2+^ buffering integrates metabolism, signaling propagation, and responses to stress or injury conditions represent active areas of research. Lastly, understanding how compartmentalized or microdomain Ca^2+^ signals in astrocyte processes regulate communication with neuronal synapses or neurovascular units—and the role buffering plays in this spatial specificity—are questions driving both experimental and computational modeling innovation (Denizot et al. 2019; Thapaliya et al. 2023).

From a computational modeling perspective, approaches to Ca^2+^ buffering can be broadly divided into implicit and explicit frameworks. Explicit models incorporate mass‐action kinetics for buffer binding and, where relevant, simulate buffer diffusion to capture the fine spatiotemporal complexity of astrocyte Ca^2+^ signaling, offering improved physiological fidelity compared to simpler, lumped‐parameter models (Denizot et al. 2019). However, even the most advanced astrocyte models tend to simplify the biological landscape, typically by pooling buffer types and neglecting the compartmental heterogeneity or regulatory adaptations known to exist in vivo. Most computational studies focus only on cytosolic buffering, often sidelining significant contributions from organellar (such as ER and mitochondria) or extracellular buffering systems, despite robust experimental evidence for their pivotal roles in shaping Ca^2+^ signals and astrocyte physiology. This simplification is further complicated by the experimental variance in buffer properties—differences in species, brain regions, cellular microenvironments, and quantification methods all contribute to divergent empirical measurements of buffer capacity and Ca^2+^ dynamics (see Table 1). Additionally, the modeling of inter‐astrocyte Ca^2+^ wave propagation, which occurs through gap‐junctional coupling in the syncytium, is an ongoing challenge: accurately reproducing such multicellular connectivity demands computational strategies that consider both local and global buffering environments (Giaume and Venance 1998; Höfer et al. 2001; Leybaert and Sanderson 2012).

Significant gaps remain in most current computational astrocyte models, impacting both their physiological accuracy and predictive capabilities. Typically, these models include only one or two types of buffers—often just mobile or immobile, but rarely a detailed spectrum that reflects physiological conditions—and usually represent kinetics using simple mass‐action laws. The diversity of endogenous buffers, such as astrocyte‐enriched proteins like CaM, parvalbumin, or S100β, along with their unique binding affinities and regulatory behaviors, is often overlooked. The spatial microanatomy of buffer localization across the extensive astrocytic arbor, which recent advances in super‐resolution imaging and particle‐based modeling highlight as essential for shaping local signaling, remains underrepresented. Furthermore, buffer concentrations and kinetics are often assumed to be static, which does not account for changes that occur during signaling plasticity, metabolic shifts, or disease progression—factors well‐documented in studies of neurodegeneration and reactive astrogliosis. Similarly, while some models incorporate fluorescent indicator dyes or genetically encoded Ca^2+^ sensors as external buffers, they rarely quantify or adjust for the complex interactions between these tools and native signaling systems, risking misinterpretation of experimental and simulated results. Finally, Ca^2+^ buffering is often modeled in isolation, with little integration with other signaling pathways (e.g., IP_3_, cAMP), metabolic regulators, or biophysical mechanisms, such as astrocyte swelling and volume regulation, despite strong evidence of their interconnectedness in the living brain. Overcoming these limitations is crucial for developing next‐generation models that accurately reflect the physiological and pathological diversity of astrocyte Ca^2+^ handling.

Crucially, the clinical and translational relevance of astrocytic Ca^2+^ buffering remains an open question. Aberrant buffer expression and function are implicated in several neurological conditions, including neurodegenerative diseases, epilepsy, and stroke (Bancroft and Srinivasan 2022; Shigetomi et al. 2016; Verkhratsky et al. 2019). Yet the precise role of buffer dysregulation—whether as a driver, modulator, or consequence of pathology—requires further exploration, including in vivo and human as well as computational studies. In addition, the growing appreciation for region‐ and age‐dependent variation in buffer protein expression, such as the age‐related decline in calreticulin in astrocytic endfeet (Zarate et al. 2021), underscores the need to incorporate such biological details into future experimental designs and models.

In conclusion, a comprehensive understanding of Ca^2+^ buffering in astrocytes is pivotal for accurate experimental analysis and predictive, mechanistic modeling. Future research should focus on incorporating buffer diversity, mobility, competition, and regulation—alongside realistic cell geometry and metabolic coupling—across various physiological and pathological contexts. Only such holistic approaches will allow the field to fully understand the significance of astrocytic Ca^2+^ buffering for neural health and pathology.

## Author Contributions

**Kerstin Lenk:** conceptualization, formal analysis, methodology, resources, visualization, writing – original draft, writing – review and editing. **Andre Zeug:** conceptualization, formal analysis, methodology, resources, visualization, writing – original draft, writing – review and editing. **Franziska E. Müller:** conceptualization, formal analysis, methodology, resources, visualization, writing – original draft, writing – review and editing.

## Funding

K.L.'s research was partially funded by the Austrian Science Fund (FWF) (10.55776/PIN7030123). This study was supported by the German Research Foundation (DFG; ZE994 to A.Z.) and by the Federal Ministry of Research, Technology and Space (BMFTR, 13N17513 to A.Z.). F.E.M. was supported by Hannover Medical School programs HiLFI and PREPARE as well as the Förderstiftung MHH plus. Funding sources were not involved in the study design, data collection, analysis, or interpretation, or in the decision to submit the article for publication.

## Conflicts of Interest

The authors declare no conflicts of interest.

## Data Availability

The authors have nothing to report.
